# Longitudinal relationship between posttraumatic cognitions and internalising symptoms in children and adolescents

**DOI:** 10.1080/20008066.2024.2398357

**Published:** 2024-10-01

**Authors:** Anke de Haan, Kristian Kleinke, Eve Degen, Markus A. Landolt

**Affiliations:** a Mental Health Research and Treatment Center (FBZ), Department of Clinical Child and Adolescent Psychology, Ruhr University Bochum, Bochum, Germany; b Department of Psychology, Division of Child and Adolescent Health Psychology, University of Zurich, Zurich, Switzerland; c Department of Psychosomatics and Psychiatry, University Children’s Hospital Zurich, Zurich, Switzerland; d Department of Psychology, University of Siegen, Siegen, Germany

**Keywords:** Adolescents, burns, children, cross-lagged panel analysis, posttraumatic cognitions, posttraumatic stress, road traffic accidents, trauma, Adolescentes, quemaduras, niños, análisis de panel cruzado, cogniciones postraumáticas, estrés postraumático, accidentes de tráfico, trauma

## Abstract

**Background:** Little is known about the naturalistic course of posttraumatic cognitions (PTCs) after exposure to a potentially traumatic event (PTE) in children and adolescents. Moreover, previous studies on the longitudinal associations of PTCs with internalising symptoms yielded mixed results.

**Objective:** To explore the naturalistic courses and longitudinal associations of dysfunctional PTCs and functional PTCs with posttraumatic stress symptoms (PTSS), depression, and anxiety.

**Method:** A total of 115 children and adolescents, aged 7–15 years, were assessed within 1 month, 3 months, and 6 months after exposure to an acute accidental PTE. Repeated measures analyses of variance were conducted to capture the naturalistic courses of PTCs and internalising symptoms. Cross-lagged panel analyses were applied to explore the longitudinal relationship between dysfunctional and functional PTCs, along with their longitudinal associations with PTSS, depression, and anxiety.

**Results:** Dysfunctional PTCs and internalising symptoms decreased, whereas functional PTCs increased over time. Dysfunctional and functional PTCs were moderately inversely related, but no significant cross-lagged paths emerged among them. Dysfunctional PTCs were moderately to strongly associated with internalising symptoms, while functional PTCs were weakly to moderately inversely associated with internalising symptoms. Initial PTSS predicted later dysfunctional PTCs (β = .31, *p *< .05), but not vice versa.

**Conclusions:** Dysfunctional PTCs, functional PTCs, and internalising symptoms were entangled over time. Our findings support the cognitive scar model with initial PTSS predicting later dysfunctional PTCs. Future research complementing between-subject with within-subject analyses could offer additional insights into the longitudinal relationship between dysfunctional PTCs, functional PTCs, and psychological symptoms.

## HIGHLIGHTS

1. Dysfunctional posttraumatic cognitions and internalising symptoms naturally decreased, whereas functional posttraumatic cognitions naturally increased over a 6-month period following exposure to an acute accidental potentially traumatic event.
2. Above the significant longitudinal associations of dysfunctional posttraumatic cognitions with internalising symptoms, functional posttraumatic cognitions were significantly inversely related to internalising symptoms over time.
3. Posttraumatic stress symptoms assessed within 1 month after the potentially traumatic event predicted dysfunctional posttraumatic cognitions assessed 3 months after the potentially traumatic event, but not vice versa.

## Abstract (es)

**Antecedentes:** Se sabe poco sobre el curso natural de las cogniciones postraumáticas (PTCs por sus siglas en inglés) después de la exposición a un evento potencialmente traumático (PTE por sus siglas en inglés) en niños y adolescentes. Además, estudios previos sobre las asociaciones longitudinales de las PTCs con los síntomas internalizantes han arrojado resultados mixtos.

**Objetivo:** Explorar el curso natural y las asociaciones longitudinales de las PTCs disfuncionales y funcionales con los síntomas de estrés postraumático (PTSS por sus siglas en inglés), depresión y ansiedad.

**Método:** Un total de 115 niños y adolescentes, de entre 7 y 15 años, fueron evaluados a 1, 3 y 6 meses después de la exposición a un PTE accidental agudo. Se realizaron análisis de medidas repetidas de varianza para capturar los cursos naturales de las PTCs y los síntomas internalizantes. Se aplicaron análisis de panel cruzado para explorar la relación longitudinal entre las PTCs disfuncionales y funcionales, junto con sus asociaciones longitudinales con los PTSS, la depresión y ansiedad.

**Resultados:** Las PTCs disfuncionales y los síntomas internalizantes disminuyeron, mientras que las PTCs funcionales aumentaron con el tiempo. Las PTCs disfuncionales y funcionales mostraron una relación moderadamente inversa, pero no surgieron rutas con rezagos cruzados significativas entre ellas. Las PTCs disfuncionales se asociaron de manera moderada a fuerte con los síntomas internalizantes, mientras que las PTCs funcionales se asociaron de manera inversamente débil a moderada con los síntomas internalizantes. Los PTSS iniciales predijeron PTCs disfuncionales posteriores (β = 0.31, *p* < .05), pero no viceversa.

**Conclusiones:** Las PTCs disfuncionales, las PTCs funcionales y los síntomas internalizantes se entrelazaron con el tiempo. Nuestros hallazgos respaldan el modelo de cicatriz cognitiva, donde los PTSS iniciales predicen PTCs disfuncionales posteriores. Investigaciones futuras que complementen análisis intersujetos con análisis intrasujetos podrían ofrecer perspectivas adicionales sobre la relación longitudinal entre las PTCs disfuncionales, las PTCs funcionales y los síntomas psicológicos.

## 1. Background

Studies among child, adolescent, and adult populations after exposure to potentially traumatic events (PTEs) display four common trajectories: resilient, recovery, delayed, and chronic (Galatzer-Levy et al., 2018). Bonanno (2004) defines *resilient* as a stable trajectory of healthy functioning across time, *recovery* as a trajectory of threshold or sub-threshold psychopathology and then a gradual return to pre-event levels, *delayed* as a trajectory of delayed trauma reactions with a delayed disruption in functioning, and *chronic* as a trajectory of chronic symptom profiles with a chronic disruption in functioning. Most studies exploring trajectories after PTEs have focused on posttraumatic stress disorder (PTSD), followed by depression, and general distress/well-being (Galatzer-Levy et al., 2018). Little is known about the naturalistic course (observation without intervention) of posttraumatic cognitions (PTCs), although dysfunctional PTCs have consistently been found to be highly correlated with trauma-related psychopathology (Gómez de La Cuesta et al., 2019) and to serve as mediators of treatment-change (Knutsen et al., 2018). Dysfunctional PTCs mean that the traumatic event and its consequences are appraised extremely negatively which maintains a feeling of current threat. As a result, short-term dysfunctional coping behaviours prevent cognitive change and cause the symptoms to persist in the long term (Ehlers & Clark, 2000). Dysfunctional PTCs comprise thoughts about the self (I am an incompetent person; I will never be the same again) and about the world (the world is a scary place where I am highly vulnerable). The role of dysfunctional PTCs in the aftermath of trauma appears as relevant in children and adolescents as previously established in adults (Gómez de La Cuesta et al., 2019). Moreover, a meta-analysis on potential risk factors for PTSD in children and adolescents reported that subjective peri-trauma factors (e.g. perceived life threat, peri-trauma fear) and post-event factors (e.g. comorbid psychological problems, thought suppression) significantly influenced the likelihood of developing PTSD following exposure to a PTE (Trickey et al., 2012). Hence, exploring the naturalistic course of PTCs in children and adolescents is a critical contribution to existing research.

Across the age groups, research on functional/adaptive ways of appraising the PTE and its consequences has been many-faceted spanning constructs such as posttraumatic growth, resilience, coping self-efficacy (CSE), and adaptive appraisals. Bosmans and van der Velden (2017), for example, reported in an adult sample exposed to trauma that trauma-related CSE negatively predicted posttraumatic stress symptoms (PTSS) over time but not vice versa. Another study in children and adolescents recruited from the hospital after single incident trauma found that adaptive appraisals were inversely related to PTSS (Hitchcock et al., 2015). Functional PTCs/adaptive appraisals might serve as a protective factor for trauma-related psychopathology. However, it is also possible that, in line with the weakest link hypothesis (Abela & Sarin, 2002), dysfunctional PTCs outweigh the protective effect of functional PTCs (Hitchcock et al., 2015). The weakest link hypothesis emerged from depression research in children and adolescents and suggests that ‘a chain is only as strong as its weakest link’ (Abela & Sarin, 2002, *p.* 815). According to this perspective, individuals’ overall vulnerability to depression may be accounted for by the most negative cognitive characteristic; and a person’s average vulnerability or their greatest strength would be less relevant in the prediction of depression (Reilly et al., 2012). More longitudinal research is needed to better disentangle the relationship of *dysfunctional* PTCs and *functional/adaptive* ways of appraising the traumatic event and its consequences over time as well as their longitudinal associations with (trauma-related) psychopathology.

Trauma researchers have applied two theoretical models that were initially developed in depression research to shed light on the timing of dysfunctional PTCs and PTSS. The *cognitive vulnerability model* suggests that depression can be explained by cognitive vulnerability (Hankin & Abramson, 2002), whereas in the *cognitive scar model* preceding depression adversely impacts cognitions (Lewinsohn et al., 1981). Research in children and adolescents exploring these two models led to mixed results. The studies conducted by Palosaari et al. (2013; involving war-affected participants) and Meiser-Stedman et al. (2019; recruitment at an emergency department following a single trauma) provided support for the cognitive vulnerability model. They demonstrated that dysfunctional PTCs predicted later PTSS, but not vice versa. Conversely, the study by de Haan et al. (2019; with participants exposed to maltreatment) reported that PTSS predicted later dysfunctional PTCs but not vice versa, supporting the cognitive scar model. Notably, using an adult sample exposed to various single trauma, Shahar et al. (2013) found evidence supporting both the cognitive vulnerability and the cognitive scar model. This was evident when examining the specific components of dysfunctional PTCs, namely dysfunctional PTCs about the self and dysfunctional PTCs about the world. Discrepant findings with respect to the cognitive vulnerability model vs. the cognitive scar model might stem from differences in samples, measures, time since the PTE, and time gaps between assessments (de Haan et al., 2019; Dekel et al., 2013; Palosaari et al., 2013; Shahar et al., 2013). No clear pattern has been identified yet.

In addition to a strong association with PTSS, dysfunctional PTCs have repeatedly been found to be highly correlated with internalising symptoms, such as depression and anxiety in children and adolescents (e.g. de Haan et al., 2016). Previous studies on cross-lagged associations of dysfunctional PTCs and PTSS in children and adolescents therefore extended their models by additionally including comorbid internalising symptoms (de Haan et al., 2019; Palosaari et al., 2016).

Considering the research gaps outlined above, our aims were threefold:

1. To explore the naturalistic course of dysfunctional PTCs and functional PTCs
2. To explore the relationship between dysfunctional and functional PTCs over time
3. To explore the longitudinal associations of dysfunctional and functional PTCs with internalising symptoms (PTSS, depression, anxiety)

Due to the mixed and little prior findings, we did not have a priori hypotheses and all our analyses were exploratory.

## 2. Method

### 2.1. Participants and procedure

School-aged patients and their parents participated in the longitudinal study *Dysfunctional Posttraumatic Cognitions in Children and Adolescents* (NCT02693249) funded by the Swiss National Science Foundation (Grant 100019_162661). The study was approved by the ethics committee of the state of Zurich, Switzerland. Inclusion criteria for the study were (a) an in- or outpatient treatment at the University Children’s Hospital Zurich, (b) an acute exposure to either a road traffic accident or a burn injury, (c) an age between 7 and 18 years, (d) written informed consent of a legal guardian and of adolescents aged 14 years and older. We selected these PTEs (road traffic accident, burn injury) a-priori since they are representative for accidental single-incident PTEs in the literature and therefore comparable to prior studies, highly prevalent in children and adolescents, and often require physical treatment or observation at a hospital which would enable us to recruit them right after the accident and follow them from the acute to chronic phase. Exclusion criteria were (a) a pervasive developmental disorder, (b) a severe head injury (initial Glasgow Coma Scale < 9), (c) an insufficient command of the German language, or (d) the accident did not meet pre-defined criteria for a road traffic accident or burn injury (e.g. smaller bicycle collision during playtime; only the hair was singed during a burn accident). Consecutive recruitment took place between February 2016 and March 2018. During this time period, 267 patients and their parents were contacted either in person, via phone, or by post. Study information and consent forms were provided in written form and in age-appropriate wording for the 11- to 18-year-olds. In total, 130 (48.7%) families consented to participate, whereas 137 (51.3%) declined. The main reasons for nonparticipation were lack of interest (*n *= 64, 46.7%), lack of time (*n *= 22, 16.1%), or that they could not be reached (*n *= 22, 16.1%). Participants and nonparticipants did not differ in regard to age, sex, type of accident, hospitalisation (yes vs. no), and days in hospital, but participants had on average a significantly higher injury severity score (ISS) compared to nonparticipants [*t*(213.93) = 2.575, *p *= .011, |*d*| = 0.32]. Notably, both participants and nonparticipants reported on average minor injury severities (participants *M *= 3.14, *SD *= 3.67; non-participants *M *= 2.17, *SD *= 2.28), keeping in mind that the ISS has a possible range between 0 and 75.

For this longitudinal analysis, 15 participants were excluded because they either did not provide data (*n *= 3), provided questionably valid data (*n *= 4), or they reported that the acute accident was not the most stressful PTE at the first assessment (*n *= 8). This led to a final sample size of 115 participants. The assessments generally took place within 1 month (*M *= 18.30, *SD *= 7.58, range = 6-43 days), 3 months (*M *= 99.58, *SD *= 10.35, range = 79-141 days), and 6 months (*M *= 193.13, *SD *= 12.73, range = 156-244 days) after the PTE.

### 2.2. Measures

Assessments with the children and adolescents were conducted in the form of a structured diagnostic interview by trained assessors, either at the participant’s home or at the University Children’s Hospital Zurich. This way, problems of understanding or random answering patterns could be detected and immediately dealt with. We used well-established validated self-report measures.

*Dysfunctional PTCs* related to the accident were assessed with the Child Post-Traumatic Cognitions Inventory (CPTCI; de Haan et al., 2016; Meiser-Stedman et al., 2009) consisting of 25 items divided into two subscales: ‘permanent and disturbing change’ (CPTCI-PC) and ‘fragile person in a scary world’ (CPTCI-SW). Examples are ‘My reactions since the frightening event mean I have changed for the worse’ (CPTCI-PC) or ‘I cannot stop bad things from happening to me’ (CPTCI–SW). Items are rated on a 4-point Likert scale from 1 (*don’t agree at all*) to 4 (*agree a lot*). The German version of the CPTCI showed good convergent validity with self-reported PTSS, depression, and anxiety, and discriminative validity between participants with and without a PTSD diagnosis rated by a clinician (de Haan et al., 2016). In the present study, Cronbach's alphas for the total score were .81 at T1, .79 at T2, and .54 at T3. For subscale CPTCI-PC, Cronbach's alphas were .78 at T1, .77 at T2, and .07 at T3. For subscale CPTCI-SW, Cronbach's alphas were .70 at T1, .71 at T2, and .59 at T3. The CPTCI subscales at T3 were not used in this study.

*Functional PTCs* related to the accident were assessed with the Functional Posttraumatic Cognitions Questionnaire (FPTCQ; de Haan et al., 2021) consisting of 11 items that cover functional PTCs related to accepting that the PTE has happened, realistically evaluating the PTE and its consequences, and continuing one’s life. Examples are ‘Although this happened to me, I can have a good life’, and ‘Although this happened to me, I am okay as a person’. The items are rated on a 4-point Likert scale with 0 (*don’t agree at all*) to 3 (*agree a lot*). The FPTCQ displayed construct validity with meaningful negative correlations with self-reported PTSS and anxiety. Importantly, functional PTCs were only moderately inversely related to dysfunctional PTCs, which suggests that the FPTCQ expands the CPTCI’s concept of permanent and disturbing change and fragile person in a scary world (de Haan et al., 2021). In the present study, Cronbach’s alphas for the total score were .75 at T1, .62 at T2, and .66 at T3.

*PTSS* and *trauma history* were assessed with the University of California at Los Angeles Post-Traumatic Stress Disorder Reaction Index for Children/Adolescents DSM–5 version (UCLA-RI-5; Landolt, 2014; Pynoos & Steinberg, 2013). For the trauma history, 14 types of PTEs were administered to the child. If more than one PTE was reported, the child was asked to identify the worst or most upsetting event. This event was referred to when assessing PTSS. PTSS were assessed by 27 items rated on a 5-point Likert scale from 0 (*none of the time*) to 4 (*most of the time*). In the present study, Cronbach’s alphas for the total symptom score were .83 at T1, .83 at T2, and .77 at T3.

*Depressive symptoms* were assessed with the Children’s Depression Inventory (CDI; Kovacs, 1985; Stiensmeier-Pelster et al., 2014) consisting of 29 items, for which the participant chooses between three statements, coded between 0 and 2. An example of the three options is ‘I am sad once in a while’, ‘I am sad many times’, ‘I am sad all the time’. In the present study, Cronbach’s alphas for the total score were .83 at T1, .85 at T2, and .79 at T3.

*Anxiety-related symptoms* were assessed with the 41 items of the Screen for Child Anxiety Related Emotional Disorders (SCARED; Birmaher et al., 1999; Steinhausen, 2005). The items are rated on a 3-point Likert scale from 0 (*not true or hardly ever true*) to 2 (*very true or often true*). The measure covers symptoms from panic disorder or significant somatic symptoms, generalised anxiety disorder, separation anxiety, social anxiety, and significant school avoidance. In the present study, Cronbach’s alphas for the total score were .90 at T1, .89 at T2, and .87 at T3.

*Sociodemographic data* were provided by the caregiver(s) in written form*.* Caregiver(s) indicated the parental education level and whether their family had experienced any of 12 significant events listed (e.g. divorce, unemployment, relocation) in the last 12 months (Landolt & Vollrath, 1998).

*Medical data* such as hospitalisation, Glasgow Coma Scale at admission, and injury severity were obtained from patient records of the University Children’s Hospital Zurich. The calculation of the injury severity score (ISS) is a two-step procedure. For each body area, only the most severe injury ranging from 1 (*minor*) to 5 (*critical*) is considered. The scores for the three most severely injured body areas are squared and summed, resulting in the ISS total score with a possible range from 0 to 75 (Baker et al., 1974).

### 2.3. Data analysis

Statistical significance was established at an α level of .05. Means, standard deviations, ranges, within- and cross-wave pairwise correlations of PTCs and internalising symptoms were calculated. To explore the naturalistic courses of PTCs and internalising symptoms, repeated measures analyses of variance (ANOVAs) were conducted over the three timepoints.

Cross-lagged panel models were applied to analyse the relationship between dysfunctional and functional PTCs over time and their associations with internalising symptoms. We followed Orth and colleagues’ (2021) recommendations and chose a-priori the traditional cross-lagged panel model (CLPM) since our focus was on between-person effects. The conceptual meaning of the cross-lagged coefficient is the ‘prospective effect of individual differences in construct X on change in individual differences in construct Y’ (Orth et al., 2021, p. 1017). The CLPM tests for change in individual differences, because the cross-lagged effects are controlled for the autoregressive effects (Orth et al., 2021). An example related to our research questions would be: When individuals have high levels of dysfunctional PTCs (relative to others), they will experience a subsequent rank-order increase in PTSS compared to individuals with low levels of dysfunctional PTCs. Using the traditional CLPM, stability was defined as relative stability, which means that relative differences among individuals on outcomes remained stable (i.e. the rank order between the participants stayed the same; Santor et al., 1997). For example, the individual who had the highest level of dysfunctional PTCs compared to the other participants at the first assessment still had the highest level of dysfunctional PTCs compared to the other participants at the second assessment.

Model 1 included dysfunctional PTCs and functional PTCs; model 2 consisted of dysfunctional PTCs, functional PTCs, PTSS, depression, and anxiety. Age, sex, trauma history, and hospitalisation were included as covariates. Since parametric modelling assumptions were violated to some degree, we obtained Yuan-Bentler test statistics, and standard errors via bootstrap (using 1000 replications). We deemed model fit acceptable, with chi-square *p* value ≥ .01, root mean square error of approximation (RMSEA) ≤ .08, standardised root mean square residual (SRMR) ≤ .10, and comparative fit index (CFI) and Tucker–Lewis index (TLI) both ≥ .95 (Schermelleh-Engel et al., 2003). Our initial aim was to conduct cross-lagged panel analyses over the 6-month period, but the model fit indices using all three assessments were inadequate. In line with previous studies (Hiller et al., 2016; Meiser-Stedman et al., 2019), we identified the timeframe from within 1 month to 3 months after the PTE to be especially relevant to explore the interplay of PTCs and internalising symptoms and therefore chose these two assessments for the cross-lagged panel analyses. We provide a stepwise setup of the cross-lagged panel models (model PTCs and PTSS; model PTCs, PTSS, and depression) in the supplementary material S2. As a sensitivity check, and comparable to Shahar et al. (2013), each cross-lagged panel model was repeated applying the specific components of dysfunctional PTCs in the form of the CPTCI subscales ‘permanent and disturbing change’ (CPTCI-PC) and ‘fragile person in a scary world’ (CPTCI-SW) (supplementary material S3). We report standardised coefficients (β) across all cross-lagged panel results.

### 2.4. Missing data

Minor cases of item nonresponse were handled by filling in the subscales’ mean values, when only up to 20% of the respective items were missing. If more than 20% was missing, these participants were not included in the respective analyses that applied pairwise and listwise deletion methods. Table S1 in the Supplementary Materials provides an overview about the available data per outcome across all assessments. For all outcomes, we had between 90.4% and 95.4% of the data available across the waves. Pairwise deletion was applied for within-wave and cross-wave pairwise correlations leading to samples sizes between 95 and 110. Listwise deletion per outcome was applied for the repeated measures ANOVAs leading to sample sizes between 94 and 104. All 115 participants were considered for the cross-lagged panel calculations, including those with missing data for single measures or time points. Full information maximum likelihood estimation was used to include all available information. Little's MCAR test including all outcome variables emerged significant, we therefore completed a comprehensive missing analysis to better understand the missing data pattern. First, we conducted bivariate correlation analyses for the binary missing data indicators of the outcome variables across time related to potential covariates and demographics. Substantial correlations with the missing data indicators would suggest deviations from the MCAR assumption. Most correlations were negligible (*r *< .1). The largest correlations were in the ‘small’ spectrum of around *r *= .2, which suggests that significant biases due to violations of MCAR are not to be expected. Second, we modelled nonresponse probabilities using logit models, where we predicted missingness in our outcome variables at the second and third assessments by observed information from the first assessment, as well as by the covariates age, sex, trauma history, and hospitalisation. The only significant relationship emerged for sex with depression and anxiety at the third assessment. Third, we conducted mixed models with maximum likelihood estimation and controlled for sex as sensitivity checks for the repeated measures ANOVAs, with similar results. To sum up, we had little missing data and rather negligible not completely random missing data patterns, and therefore expect the reported results to be unbiased.

We used SPSS software 27.0 for descriptives, pairwise correlations, and repeated measures ANOVAs. Cross-lagged panel results were obtained using R 4.3.2 running under Windows 11 x64 with package lavaan_0.6-17 (Rosseel, 2012). The R script is provided as supplementary material.

## 3. Results

### 3.1. Descriptives

Table 1 displays study characteristics. Around 71% of the school-aged participants had experienced an acute road traffic accident and around 29% an acute burn injury. Almost half of the participants stayed at least one night at the hospital for treatment and/or monitoring. Three participants (2.6%) received counselling/therapy within the 6-month period after the accident. Table 2 gives on overview of the PTCs and internalising symptoms at the different assessments. Less than 15% of the sample showed clinically relevant dysfunctional PTCs and internalising symptoms at one of the timepoints (for the specific cut-offs see Table 2). Table 3 displays the within- and cross-wave pairwise correlations of PTCs and internalising symptoms. Functional PTCs were inversely related to dysfunctional PTCs and internalising symptoms across all assessments.

**Table 1.** Sociodemographic data and characteristics of the potentially traumatic event.

Variables	Group or range		*n* or *M*	% or *SD*
Age at accident*	Children (7–10 years)		51	44.3
	Adolescents (11–15 years)		64	55.7
Sex	Male		62	53.9
	Female		53	46.1
Maternal education	University		29	25.2
	A-level equivalent or college of higher education		19	16.5
	Completed apprenticeship (3–4 years)		25	21.7
	Completed apprenticeship (1–2 years)		24	20.9
	Completed mandatory school		4	3.5
	Mandatory school not completed		4	3.5
	Not determined+		10	8.7
Paternal education	University		35	30.4
	A-level equivalent or college of higher education		23	20.0
	Completed apprenticeship (3–4 years)		18	15.7
	Completed apprenticeship (1–2 years)		13	11.3
	Completed mandatory school		4	3.5
	Mandatory school not completed		1	0.9
	Not determined+		21	18.3
Parental education# (possible range 2–12)	2–12		8.93	2.54
Recent life events family (possible range 0–12)	0–5		1.13	1.28
Trauma history: child was exposed to other trauma type(s) in the past	Yes		51	44.3
	No		64	55.7
Potentially traumatic event	Road traffic accident		82	71.3
	Burn injury		33	28.7
Injury severity score (possible range 0–75)	0–29		3.24	3.81
Hospitalisation	No		66	57.4
	Yes		49	42.6
	Days in hospital 1–23		3.96	5.18
				

Note*.* *Total sample *M* = 11.09, *SD* = 2.41. +Participants could not be reliably classified in any category due to insufficient information. #Sum of maternal education and paternal education. In case only information for one parent was available, their score was included twice in the parental education score.

**Table 2.** Description of posttraumatic cognitions and internalising symptoms over time.

Outcome (possible range; clinical cut-off)	1st assessment (within 1 month after the potentially traumatic event)				2nd assessment (3 months after the potentially traumatic event)				3rd assessment (6 months after the potentially traumatic event)			
	*M*	*SD*	Range	% above clinical cut-off	*M*	*SD*	Range	% above clinical cut-off	*M*	*SD*	Range	% above clinical cut-off
Dysfunctional PTCs (25–100; 46)	36.36	7.81	25–58	14.5	33.46	6.84	25–64	4.7	32.36	5.52	25–53	2.7
Functional PTCs (0–33)	29.79	3.45	16–33	NA	31.70	1.85	25–33	NA	32.25	1.46	24–33	NA
PTSS (0–80; 35)	15.10	10.46	0–49	4.6	11.32	9.06	0–49	1.9	8.98	7.31	0–39	0.9
Depression (0–58; 15)	7.52	5.49	0–30	12.1	5.83	5.33	0–29	7.8	5.33	4.54	0–21	5.6
Anxiety (0-82; 25)	13.92	10.37	0–51	14.4	10.81	8.64	0–37	8.9	8.53	7.21	0–36	3.7

* *Note*.* PTCs = posttraumatic cognitions. PTSS = posttraumatic stress symptoms. Sample size per outcome and assessment between 101 and 113 participants.

**Table 3.** Within- and cross-wave pairwise correlations of posttraumatic cognitions and internalising symptoms.

		1st assessment (within 1 month after the PTE)					2nd assessment (3 months after the PTE)					3rd assessment (6 months after the PTE)				
		dPTCs	fPTCs	PTSS	Depr	Anx	dPTCs	fPTCs	PTSS	Depr	Anx	dPTCs	fPTCs	PTSS	Depr	Anx
1st	dPTCs	—	−.42***	.67***	.47***	.59***	.58***	−.35***	.41***	.35***	.49***	.49***	−.04	.23*	.28**	.36***
	fPTCs	−.42***	—	−.36***	−.22*	−.44***	−.34***	.60***	−.23*	−.20*	−.35***	−.24*	.42***	−.06	−.11	−.24*
	PTSS	.67***	−.36***	—	.48***	.71***	.56***	−.33**	.64***	.40***	.61***	.36***	−.17	.49***	.26**	.54***
	Depr	.47***	−.22*	.48***	—	.61***	.35***	−.32**	.45***	.73***	.50***	.40***	−.08	.35***	.72***	.47***
	Anx	.59***	−.44***	.71***	.61***	—	.48***	−.40***	.42***	.46***	.67***	.41***	−.19	.29**	.34**	.66***
2nd	dPTCs	.58***	−.34***	.56***	.35***	.48***	—	−.47***	.66***	.43***	.65***	.61***	−.50***	.33**	.33**	.31**
	fPTCs	−.35***	.60***	−.33**	−.32**	−.40***	−.47***	—	−.40***	−.37***	−.44***	−.45***	.59***	−.29**	−.33**	−.33**
	PTSS	.41***	−.23*	.64***	.45***	.42***	.66***	−.40***	—	.61***	.72***	.44***	−.31**	.50***	.49***	.44***
	Depr	.35***	−.20*	.40***	.73***	.46***	.43***	−.37***	.61***	—	.59***	.43***	−.10	.36***	.75***	.48***
	Anx	.49***	−.35***	.61***	.50***	.67***	.65***	−.44***	.72***	.59***	—	.37***	−.28**	.37***	.42***	.61***
3rd	dPTCs	.49***	−.24*	.36***	.40***	.41***	.61***	−.45***	.44***	.43***	.37***	—	−.28**	.45***	.46***	.38***
	fPTCs	−.04	.42***	−.17	−.08	−.19	−.50***	.59***	−.31**	−.10	−.28**	−.28**	—	−.18	−.03	−.08
	PTSS	.23*	−.06	.49***	.35***	.29**	.33**	−.29**	.50***	.36***	.37***	.45***	−.18	—	.56***	.51***
	Depr	.28**	−.11	.26**	.72***	.34**	.33**	−.33**	.49***	.75***	.42***	.46***	−.03	.56***	—	.54***
	Anx	.36***	−.24*	.54***	.47***	.66***	.31**	−.33**	.44***	.48***	.61***	.38***	−.08	.51***	.54***	—

Note*.* Anx = anxiety. Depr = depression. dPTCs = dysfunctional posttraumatic cognitions. fPTCs = functional posttraumatic cognitions. PTSS = posttraumatic stress symptoms. PTE = potentially traumatic event. Sample size per pairwaise correlation between 95 and 110 participants. ***p < .001, **p < .01, *p < .05.

### 3.2. Naturalistic course

Dysfunctional PTCs decreased *F*(1.76, 177.25) = 23.45, *p *< .001, η_p_^2 ^= 0.188; whereas functional PTCs increased over time *F*(1.39, 143.56) = 54.50, *p *< .001, η_p_^2 ^= 0.346. Pairwise comparisons (i.e. 1st vs. 2nd assessment, 1st vs. 3rd assessment, 2nd vs. 3rd assessment) yielded significant differences between each timepoint, except for dysfunctional PTCs assessed 3 months vs. 6 months after the PTE.

The internalising symptoms PTSS *F*(2, 196) = 26.95, *p *< .001, η_p_^2 ^= 0.216; depression *F*(2, 192) = 17.80, *p *< .001, η_p_^2 ^= 0.156; and anxiety *F*(2, 186) = 23.29, *p *< .001, η_p_^2 ^= 0.200 decreased over time as well. Pairwise comparisons yielded significant differences, except for depression assessed 3 months vs. 6 months after the PTE.

### 3.3. Relationship between dysfunctional and functional PTCs over time

**Figure 1.**
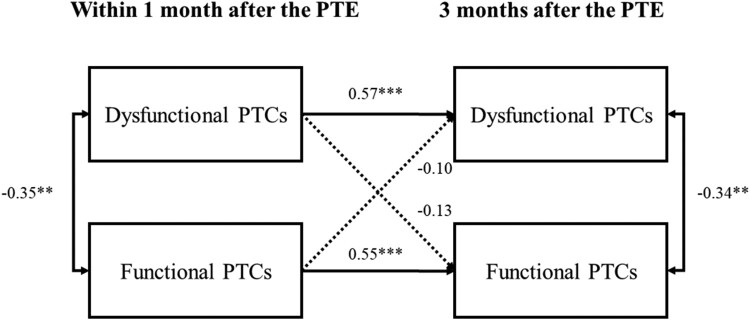
Cross-lagged panel analysis of the relationship between dysfunctional and functional posttraumatic cognitions over time. PTCs = posttraumatic cognitions. PTE = potentially traumatic event. Controlled for age, sex, trauma history, and hospitalisation. ***p < .001, **p < .01.

We found strong relative stability in both dysfunctional PTCs and functional PTCs (β = .57; β = .55; both *p *< .001, Figure 1). Dysfunctional and functional PTCs were moderately correlated at both timepoints (*r *= −.35; *r *= −.34; both *p *< .01). No significant cross-lagged path emerged (β = −.10; β = −.13; both *p *> .05). The autoregressive and cross-lagged paths accounted for 38.5% and 37.3% of variance in the dependent variables assessed 3 months after the PTE. The model fit indices indicating adequate model fit are displayed in Table 4. Only the TLI value of .920 was slightly below the cutoff of .95.

**Table 4.** Model fit indices of the cross-lagged panel models.

	Acceptable fit	*χ^2^ p* ≥ .01	*df*	CFI≥0.95	TLI≥0.95	RMSEA≤0.08	90% CI	SRMR≤0.10
Model	dPTCs, fPTCs	11.424 *p *= .179	8	0.977	0.920	0.062	0.000–0.136	0.037
	dPTCs, fPTCs, PTSS, Depr, Anx	27.871 *p *= .112	20	0.988	0.947	0.061	0.000–0.111	0.031

Note. Anx = anxiety. CFI = comparative ﬁt index. CI = confidence interval. Depr = depression. *df* = degrees of freedom. dPTCs = dysfunctional posttraumatic cognitions. fPTCs = functional posttraumatic cognitions. PTSS = posttraumatic stress symptoms. RMSEA = root mean square error of approximation. SRMR = standardised root mean square residual. TLI = Tucker Lewis index.

### 3.4. Longitudinal associations of PTCs and internalising symptoms

**Figure 2.**
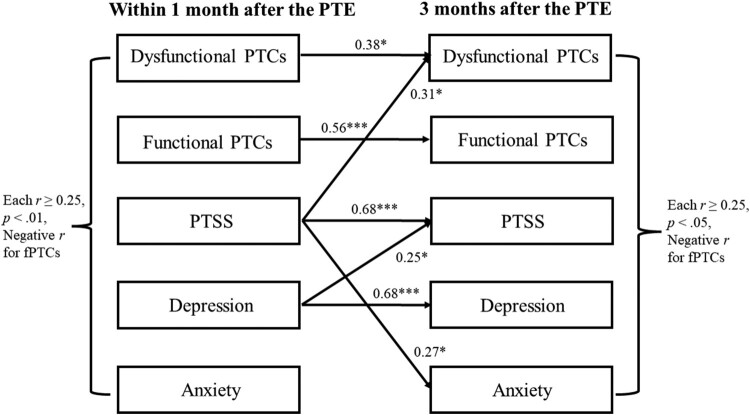
Cross-lagged panel analysis of the longitudinal associations of posttraumatic cognitions with internalising symptoms. (f)PTCs = (functional) posttraumatic cognitions. PTE = potentially traumatic event. PTSS = posttraumatic stress symptoms. Controlled for age, sex, trauma history, and hospitalisation. Only significant regression paths are shown. ***p < .001, *p < .05.

The longitudinal associations between PTCs and internalising symptoms are displayed in Figure 2. Moderate to strong relative stability emerged in PTCs and internalising symptoms (each β≥.36, *p *< .05 except for anxiety *p *= .064). Dysfunctional PTCs and internalising symptoms were moderately to strongly correlated at both timepoints (within 1 month: *r *≥ .50, *p *< .001; after 3 months: *r *≥ .29, *p *< .05). Functional PTCs were weakly to moderately inversely related to internalising symptoms (within 1 month: *r *≥ −.25, *p *< .01; after 3 months: *r *≥ −.25, *p *< .05). Significant cross-lagged paths emerged: PTSS assessed within 1 month after the PTE moderately predicted dysfunctional PTCs (β = .31, *p *< .05) and weakly predicted anxiety (β = .27, *p *< .05) assessed 3 months after the PTE. Depression assessed within 1 month after the PTE weakly predicted PTSS assessed 3 months after the PTE (β = .25, *p *< .05). The autoregressive and cross-lagged paths accounted for 40.0% to 52.2% of variance in the dependent variables assessed 3 months after the PTE.

## 4. Discussion

This longitudinal study explored the naturalistic course of PTCs (dysfunctional and functional) and internalising symptoms (PTSS, depression, anxiety) in a sample of 115 children and adolescents exposed to an acute accidental PTE. Assessments spanned from the acute phase (within 1 month after the PTE) to the chronic phase (3 and 6 months after the PTE). Research questions aimed at examining the naturalistic course of PTCs, the relationship between dysfunctional and functional PTCs over time, and their longitudinal associations with internalising symptoms (PTSS, depression, and anxiety).

Aim 1 explored the naturalistic course of PTCs. Without any interventions, we saw a decrease in dysfunctional PTCs and an increase in functional PTCs. This might support the notion that PTCs behave more like a state-like than a trait-like outcome. Differences might emerge for more severely burdened children and adolescents. However, a study by de Haan et al. (2019) looking into children and adolescents exposed to multiple maltreatment experiences also found a decline of dysfunctional PTCs over time, although the PTCs load was higher across all assessments. Notably, we did not find a significant change in dysfunctional PTCs from 3 to 6 months after the PTE which might suggest that their natural recovery plateau at some point. Hiller et al.’s (2016) conclusion that screening PTSD at 3 months after the PTE may be effective might be transferable to dysfunctional PTCs.

PTSS, depression, and anxiety also decreased over time. For most participants, the levels of dysfunctional PTCs and internalising symptoms were below the clinical cut-offs from the start. Hence, our findings align with the overview of Galatzer-Levy et al. (2018) that reported that the resilience trajectory was the modal response across studies (65.7%), followed by recovery (20.8%), chronicity (10.6%), and delayed onset (8.9%). High resilience rates have consistently been reported across trauma types and study populations (Bonanno et al., 2023). Our results also support the previous meta-analytic finding of significant natural recovery of PTSD up to 6 months after the PTE (Hiller et al., 2016).

Aim 2 explored the relationship between dysfunctional and functional PTCs. Dysfunctional and functional PTCs were moderately inversely related to each other over time. Weak and non-significant cross-lagged paths emerged among them. In examining the specific components of dysfunctional PTCs, some models revealed significant but still weak cross-lagged paths from dysfunctional PTCs of being a fragile person in a scary world assessed within 1 month to functional PTCs assessed 3 months after the PTE. Hence, in these models, more dysfunctional PTCs of being a fragile person in a scary world predicted less functional PTCs later. This finding is in line with study results by Cieslak et al. (2008) in adults exposed to an acute motor vehicle accident. The longitudinal relationship between dysfunctional and functional PTCs (moderate correlations at the respective assessments and weak predominately non-significant cross-lagged paths) suggests treating dysfunctional PTCs and functional PTCs as independent outcomes.

Aim 3 explored the longitudinal associations between PTCs and internalising symptoms. We found significant moderate to strong relative stability for PTCs and internalising symptoms. The co-occurrence of decrease over time and moderate to strong autoregressive paths might indicate that, on average, participants had the same trajectory – natural decrease of dysfunctional PTCs and internalising symptoms and natural increase of functional PTCs. However, moderate to strong effect sizes also suggest that in some cases the rank order between individual participants was changing over time. On average, there was a significant decrease/increase over time, but the extent and timing might have varied between participants. Previous studies conducting cross-lagged panel analyses on dysfunctional PTCs and PTSS in children and adolescents differed in their findings on stability parameters for dysfunctional PTCs ranging from very strong (Meiser-Stedman et al., 2019) to moderate to strong effect sizes (de Haan et al., 2019; Palosaari et al., 2013). These studies also reported mixed findings for the stability of PTSS over time, from small (Meiser-Stedman et al., 2019; Palosaari et al., 2013) to moderate effect sizes (de Haan et al., 2019). Time since trauma and time between assessments might impact the stability parameters and thus might have led to these mixed findings in previous studies. Future analyses exploring individual PTCs trajectories might help to shed more light into the temporal stability of PTCs in children and adolescents.

Dysfunctional PTCs were moderately to strongly related to internalising symptoms, whereas functional PTCs were inversely weakly to moderately associated with internalising symptoms over time. The moderate to strong relationship of dysfunctional PTCs and internalising symptoms is in line with both previous meta-analytic findings (Gómez de La Cuesta et al., 2019) and prior cross-lagged panel analyses in children and adolescents (de Haan et al., 2019; Meiser-Stedman et al., 2019; Palosaari et al., 2013). A strength of cross-lagged panel models is to simultaneously control for all associations. Functional PTCs still played a role above the associations of dysfunctional PTCs with internalising symptoms. Neither dysfunctional PTCs nor functional PTCs predicted internalising symptoms over time (this was also true when running the models without dysfunctional PTCs), we can therefore neither support nor mitigate the weakest link hypothesis. We can conclude from our findings that functional PTCs interact with internalising symptoms above the impact of dysfunctional PTCs. Future studies might gain more insight by exploring single dysfunctional and functional PTCs specifically relevant to the individual rather than applying a total score when investigating the relationship of dysfunctional and functional PTCs and their interaction (weakest link hypothesis) with psychopathology. Moreover, in addition to exploring the specific content of PTCs relevant to the individual, a predictive processing perspective by Kube et al. (2020) suggests that the aberrant precision of probabilistic prior predictions ( =  high confidence in dysfunctional PTCs) and lack of updating them should be taken into account.

We consistently found that PTSS assessed within 1 month predicted dysfunctional PTCs assessed 3 months after the PTE. This finding aligns with results from a diverse range of studies (and contradicts with a diverse range of studies; see next paragraph): de Haan et al. (2019) investigated a distinct sample of children and adolescents exposed to maltreatment, employing different assessment points with the second assessment occurring six months after the first, and the third assessment up to 18 months after the second. Similarly, a 17-year longitudinal study on Israeli combat veterans conducted by Dekel et al. (2013), as well as a study on adults with various single trauma exposures assessed at 2, 4, and 12 weeks after a PTE by Shahar et al. (2013), all reported a consistent pattern where PTSS were predictive of subsequent dysfunctional PTCs. This finding is also in line with the cognitive scar model (Lewinsohn et al., 1981) and with Ehlers’ and Clark’s (2000) suggestion that the negative interpretation of one's initial PTSS may lead to later dysfunctional PTCs. They specify that the initial PTSS may be related to later dysfunctional PTCs *about the self* (e.g. ‘My personality has changed for the worse’, ‘I'll never get over this’; *p*. 322). When looking into the specific components of dysfunctional PTCs, we did not find that initial PTSS predicted later dysfunctional PTCs of a permanent and disturbing change, but that initial PTSS predicted later dysfunctional PTCs of being a fragile person in a scary world. This difference could mean that for children and adolescents initial PTSS are more related to dysfunctional PTCs about the world than to dysfunctional PTCs about the self or this difference could be due to the fact that the content of the subscales of the adult and child versions are slightly different. Notably, Shahar et al.’s (2013) study in adults reported that initial PTSS predicted both later dysfunctional PTCs about the self and the world.

Across all cross-lagged panel analyses, irrespective of using the dysfunctional PTCs total score or its specific components, we did not find any support for the cognitive vulnerability model. Preceding dysfunctional PTCs did not predict any later internalising symptoms (PTSS, depression, or anxiety). This is in contrast to the findings by Meiser-Stedman et al. (2019), which featured a notably similar sample (school-aged participants recruited from the hospital emergency department following a single trauma) and assessment time-points (2–4 weeks and 2 months after the PTE). This discrepancy is also evident when compared to a study sample involving war-affected children assessed at 3, 5, and 11 months after the war (Palosaari et al., 2013; Palosaari et al., 2016) which both supported the cognitive vulnerability model. It is noteworthy that the previously mentioned study by Shahar et al. (2013) supported both models, indicating that dysfunctional PTCs about the self predicted subsequent PTSS, as well as PTSS predicted subsequent dysfunctional PTCs about the self and the world in an adult sample.

In summary, a definitive pattern does not emerge to favour either the cognitive vulnerability model or the cognitive scar model in trauma adjustment. It might therefore be important to consider both possibilities when planning trauma treatment. For some patients, the presence or interpretation of initial PTSS might fuel their dysfunctional PTCs and this might be important to consider early on in treatment.

We did not find any significant cross-lagged associations between functional PTCs and PTSS, depression, or anxiety. This is in contrast with Bosmans’ and van der Velden’s (2017) study on adults that found that trauma-related CSE predicted later PTSS but not vice versa in an acute trauma-exposed sample and vice versa in a sample exposed to a PTE 1–2 years before the first assessment. Notably, for both samples, the cross-lagged effects for trauma-related CSE predicting later PTSS were small (β ≤ -.16).

Although not a focus of our study but included as control variables were the longitudinal associations of internalising symptoms. Depression assessed within 1 month predicted PTSS assessed 3 months after the PTE. This is in line with previous studies in children (Palosaari et al., 2016), adolescents (Ying et al., 2012), and adults (Schindel-Allon et al., 2010) which all reported that depression predicted later PTSS but not vice versa. Schindel-Allon et al. (2010) suggest that a possible mechanism could be fear extinction. Depressive symptoms could hinder an individual’s motivation and ability to engage in exposure to trauma-related stimuli. From a clinical point of view, it supports the good clinical practice to conduct broad diagnostics to detect comorbid disorders but also (sub)clinical levels of psychopathology that might hinder the treatment process. Another significant cross-lagged path emerged for PTSS and anxiety. PTSS assessed within 1 month predicted anxiety assessed 3 months after the PTE, aligning with the clinical experience that treating PTSS first can have a beneficial effect on comorbid symptoms.

A strength of our study was conducting the assessments of PTCs and internalising symptoms in the form of a structured interview by trained assessors. This way, we could check whether the school-aged participants understood the items. Moreover, we did not only explore dysfunctional PTCs and PTSS which has repeatedly been done before but extended the analyses to functional PTCs, depression, and anxiety. Both PTCs and internalising symptoms are difficult to detect from an outside perspective, we therefore think that relying on the child report was appropriate.

Luckily, the majority of our sample dealt well with the PTE from the start. This is in line with the current state of research. However, we only explored the naturalistic course of acute accidental PTEs. Therefore, our findings might not be generalisable to interpersonal PTE and/or to children and adolescents with clinically relevant distress levels. In addition, although we did not intervene, we did interact with the participants and their parents three times. Talking about the PTE, and the associated cognitions and symptoms during the interviews might have helped the children and adolescents to better cope with their experience. From a statistical point of view, we had to deal with a floor effect in all outcomes since 85% of the sample reported PTCs and symptoms below the clinically relevant cut-offs across all timepoints. We assume that lack in variance hindered us to conduct cross-lagged panel analyses over all three timepoints. Cronbach’s alpha values for dysfunctional PTCs at the third assessment and for functional PTCs at the second and third assessments were below .70.

One of the biggest challenges in exploring the longitudinal associations between PTCs and symptoms lies in the heterogeneity of studies in terms of samples, timescales of assessments, and outcomes. This makes it incredibly difficult to draw conclusions and to explain differences in results, for example in terms of cognitive vulnerability model vs. cognitive scar model. Moreover, research studies on topics such as cognitive vulnerability model vs. cognitive scar model or the weakest link hypothesis have investigated average scores across samples, with mixed results for children, adolescents, and adults. To gain more insight into the relationship between dysfunctional and functional PTCs and their longitudinal associations with internalising (and externalising) symptoms, it seems therefore necessary that future studies complement between-subject with within-subject analyses. This is also in line with the common clinical perspective that patients and their (trauma-related) psychopathology are unique, as is treatment planning. Future studies exploring much shorter timescales (e.g. hours) would be an important addition to fully understand the reciprocal associations between PTCs and symptoms. Irrespective whether PTCs drives PTSS or vice versa, cognitive behavioural therapies with a trauma focus seem to be a reasonable choice to simultaneously address PTCs and PTSS. Moreover, as PTCs, PTSS, depression, and anxiety are entangled over time, cognitive behavioural therapies with a trauma focus efficaciously treat comorbid symptoms as well (de Haan et al., 2024). However, in line with previous findings on PTSD (Hiller et al., 2016), the screening of (dysfunctional) PTCs might be most informative around 3–6 months after the PTE and the treatment of (dysfunctional) PTCs should not interfere with a natural recovery process.

Most children and adolescents dealt well with the acute accidental PTE. Dysfunctional PTCs and internalising symptoms declined without any intervention within the 6-month period after the PTE, while functional PTCs increased. PTCs and internalising symptoms were entangled over time. PTSS assessed within 1 month predicted dysfunctional PTCs assessed 3 months after the PTE. Future research on dysfunctional and functional PTCs might benefit from exploring individual trajectories as well as single dysfunctional and functional PTCs specifically relevant to the individual rather than total PTCs scores to shed more light on the longitudinal relationship between dysfunctional PTCs, functional PTCs, and (trauma-related) psychopathology.

## Supplementary Material

Supplemental Material

## Data Availability

The data of this study are available from the corresponding author upon reasonable request.
