# Supplementary material for: Longitudinal relationship between posttraumatic cognitions and internalising symptoms in children and adolescents
Source: Eur J Psychotraumatol. 2024 Oct 1;15(1):2398357. doi: 10.1080/20008066.2024.2398357 (PMC11445896; doi:10.1080/20008066.2024.2398357)
Supplement: Supplemental Material [file ZEPT_A_2398357_SM0360.pdf]

Supplementary Materials

de Haan, Kleinke, Degen, Landolt

S1: Available data across assessments

Supplementary material S1 displays the available data per outcome across assessments.

Table S1

Available data across assessments, total sample 115 participants

**Outcome**	**1st assessment**		**2nd assessment**		**3rd assessment**		**Across all assessments**	
	n	%	*n*	%	*n*	%	*n*	%
Dysfunctional PTCs	110	95.7	106	92.2	110	95.7	326	94.5
Functional PTCs	113	98.3	106	92.2	110	95.7	329	95.4
PTSS	109	94.8	107	93.0	107	93.0	323	93.6
Depression	107	93.0	102	88.7	108	93.9	317	91.9
Anxiety	104	90.4	101	87.8	107	93.0	312	90.4

Note. PTCs = posttraumatic cognitions. PTSS = posttraumatic stress symptoms.

S2: *Stepwise setup of cross-lagged panel models*

Supplementary material S2 includes the model fit indices (Table S2.1) and the figures of the stepwise setup of the cross-lagged panel models (Figure S.2.2 Model PTCs and PTSS; Figure S.2.3 Model PTCs, PTSS, and depression).

Table S2.1

Model fit indices of the stepwise setup of the cross-lagged panel models

		χ^2	*df*	CFI	TLI	RMSEA	90% CI	SRMR
	Acceptable fit	***p≥.01***		***≥0.95***	***≥0.95***	***≤0.08***		***≤0.10***
**Model**	**dPTCs, fPTCs, PTSS**	16.597 *p*=.165	12	0.985	0.943	0.060	0.000 – 0.124	0.034
	**dPTCs, fPTCs, PTSS, Depr**	24.620 *p*=.077	16	0.980	0.917	0.073	0.000 – 0.125	0.033

Note. CFI = comparative fit index. CI = confidence interval. Depr = depression. *df* = degrees of freedom. dPTCs = dysfunctional posttraumatic cognitions. fPTCs = functional posttraumatic cognitions. PTSS = posttraumatic stress symptoms. RMSEA = root mean square error of approximation. SRMR = standardized root mean square residual. TLI = Tucker Lewis index.

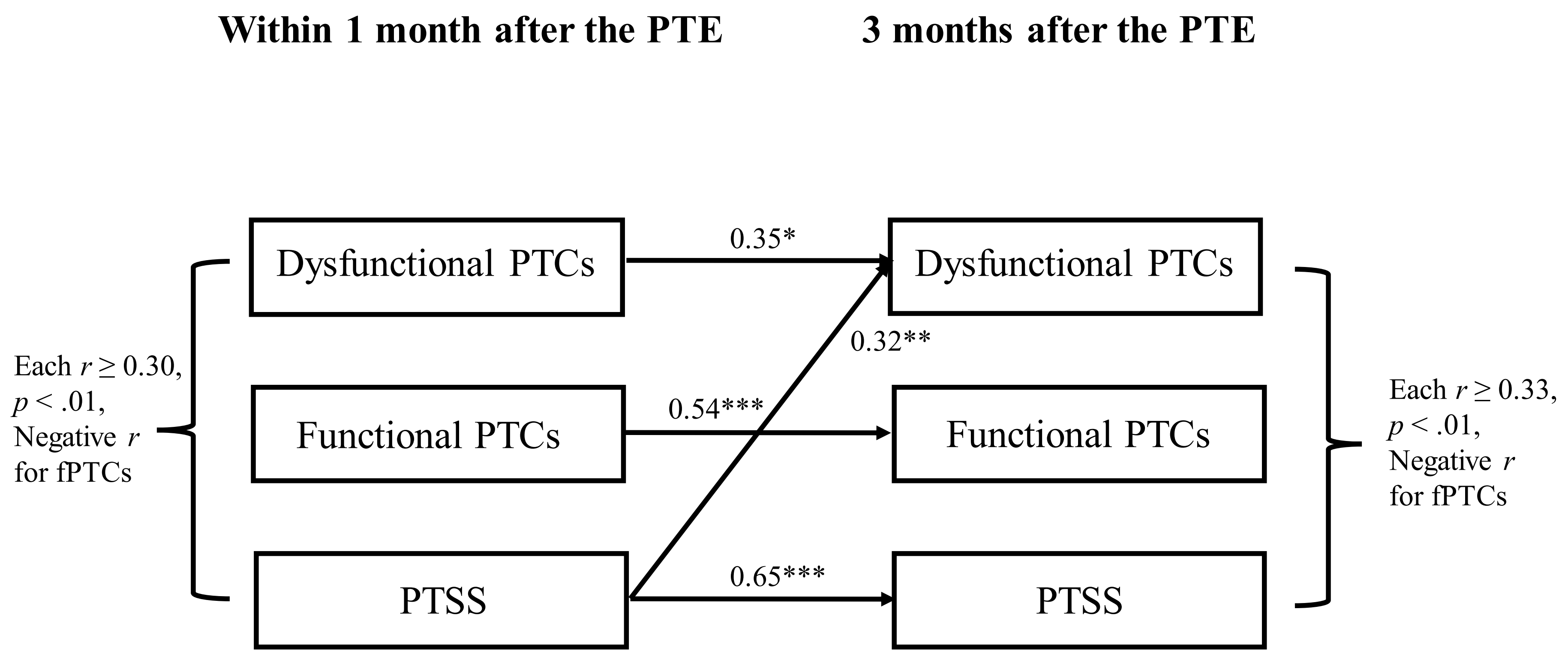

Figure S.2.2. Cross-lagged panel analysis of the longitudinal associations of posttraumatic cognitions with posttraumatic stress symptoms

(f)PTCs = (functional) posttraumatic cognitions. PTE = potentially traumatic event. PTSS = posttraumatic stress symptoms.

Controlled for age, sex, trauma history, and hospitalisation. Only significant regression paths are shown. ****p*<.001, ***p*<.01, **p*<.05.

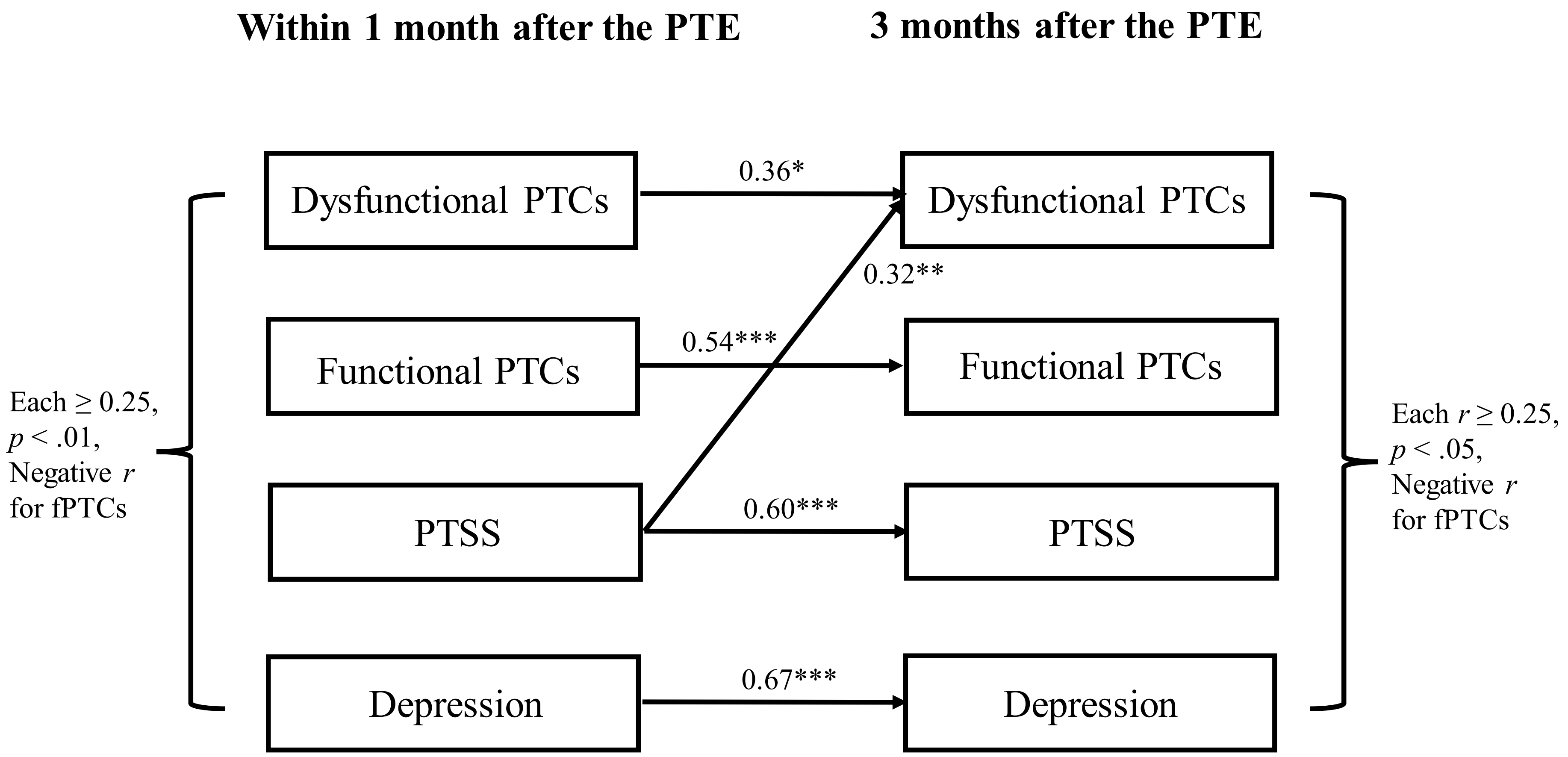

Figure S.2.3. Cross-lagged panel analysis of the longitudinal associations of posttraumatic cognitions with posttraumatic stress symptoms and depression

(f)PTCs = (functional) posttraumatic cognitions. PTE = potentially traumatic event. PTSS = posttraumatic stress symptoms.

Controlled for age, sex, trauma history, and hospitalisation. Only significant regression paths are shown. ***p<.001, **p<.01, *p<.05.

S3: *Specific components of dysfunctional posttraumatic cognitions*

Supplementary material S3 displays the model fit indices (Table S3.1) and the figures for all cross-lagged panel models using the specific components of dysfunctional PTCs of a permanent and disturbing change and of dysfunctional PTCs of being a fragile person in a scary world (Figures S3.2 to S3.5). In contrast to the analyses with the dysfunctional PTCs' total score, dysfunctional PTCs of being a fragile person in a scary world assessed within 1 month after the PTE significantly predicted functional PTCs assessed 3 months after the PTE (β=-.23, p<.01; Figure S.3.2), however, this significant but weak cross-lagged path vanished once depression and anxiety were included in the model. In line with the analyses using the dysfunctional PTCs' total score, PTSS assessed within 1 month after the PTE moderately predicted dysfunctional PTCs assessed 3 months after the PTE, but only dysfunctional PTCs of being a fragile person in a scary world (β=.33, p<.01; Figure S3.5).

Table S3.1

Model fit indices of the cross-lagged panel models exploring the specific components of dysfunctional posttraumatic cognitions

		χ^2	***df***	**CFI**	**TLI**	**RMSEA**	**90% CI**	**SRMR**
	Acceptable fit	***p≥.01***		***≥0.95***	***≥0.95***	***≤0.08***		***≤0.10***
**Model**	**dPTCs – PC SW, fPTCs**	14.838 p=.250	12	0.988	0.956	0.044	0.000-0.112	0.037
	**dPTCs – PC SW, fPTCs, PTSS**	20.701 p=.190	16	0.988	0.951	0.052	0.000-0.109	0.033
	**dPTCs – PC SW, fPTCs, PTSS, Depr**	31.374 p=.050	20	0.978	0.901	0.073	0.000-0.121	0.033
	**dPTCs – PC SW, fPTCs, PTSS, Depr, Anx**	34.709 p=.073	24	0.986	0.929	0.065	0.000-0.111	0.033

Note. Anx = anxiety. CFI = comparative fit index. CI = confidence interval. Depr = depression. *df* = degrees of freedom. dPTCs = dysfunctional posttraumatic cognitions: PC = permanent and disturbing change, SW = fragile person in a scary world. fPTCs = functional posttraumatic cognitions. PTSS = posttraumatic stress symptoms. RMSEA = root mean square error of approximation. SRMR = standardized root mean square residual. TLI = Tucker Lewis index.

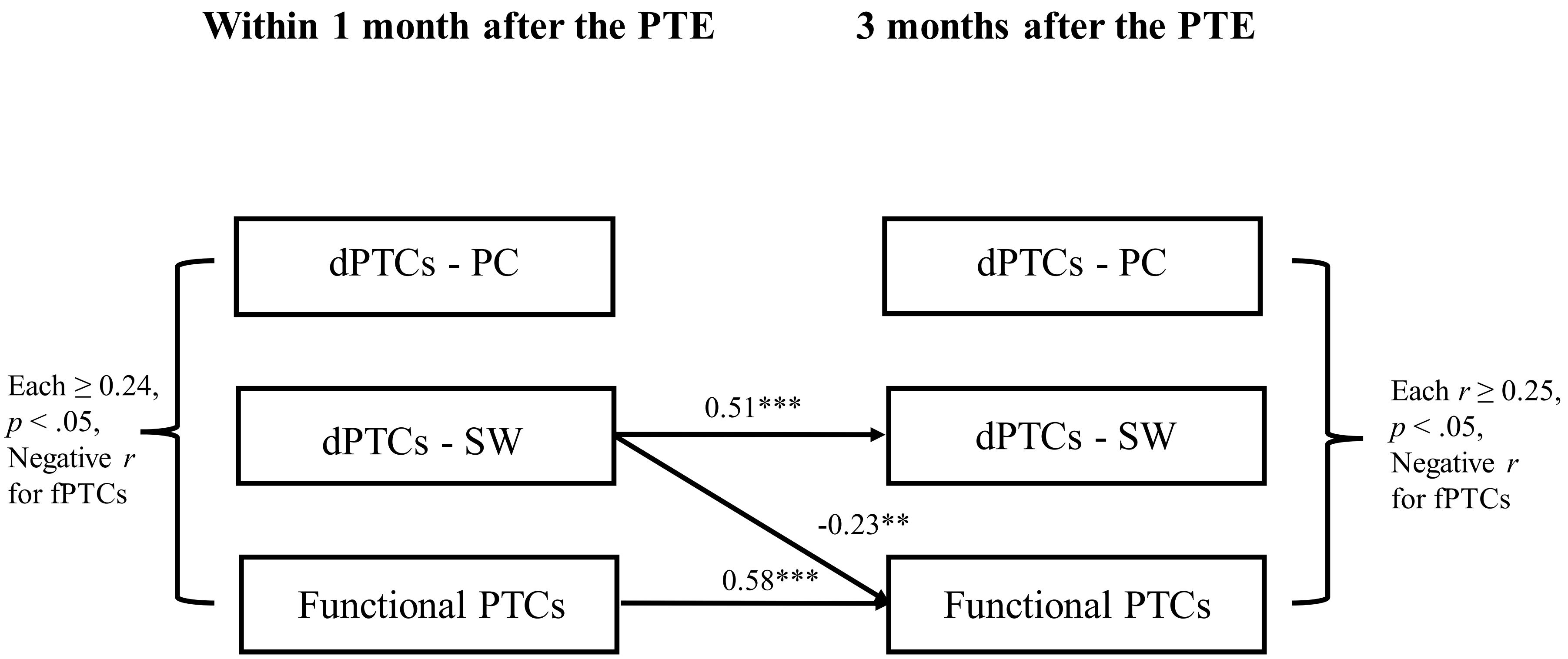

Figure S.3.2. Cross-lagged panel analysis of the relationship between dysfunctional and functional posttraumatic cognitions over time
dPTCs = dysfunctional posttraumatic cognitions: PC = permanent and disturbing change, SW = fragile person in a scary world.
fPTCs = functional posttraumatic cognitions. PTE = potentially traumatic event. Controlled for age, sex, trauma history, and hospitalisation.
Only significant regression paths are shown. ***p<.001, **p<.01.

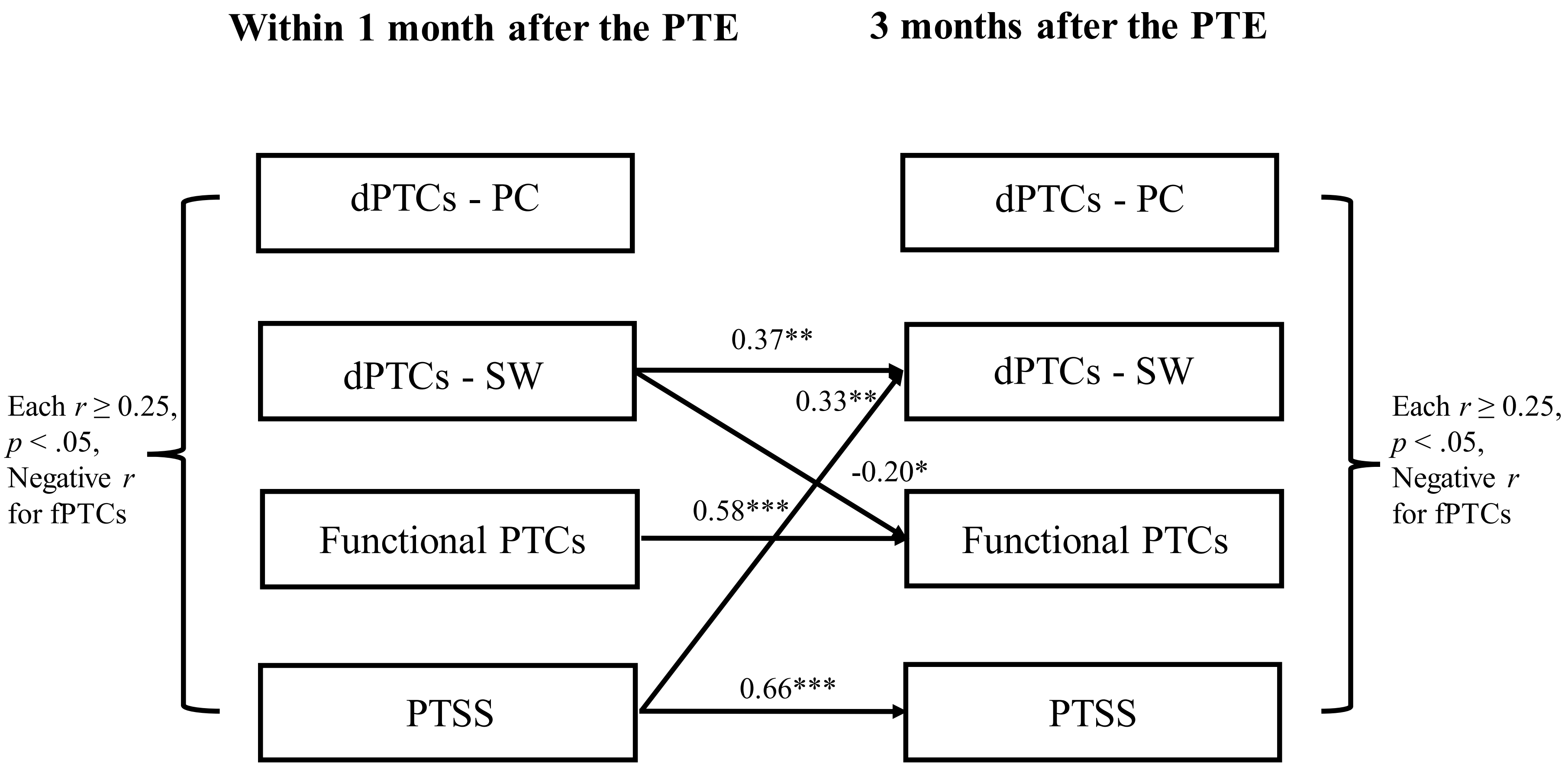

Figure S.3.3. Cross-lagged panel analysis of the longitudinal associations of posttraumatic cognitions with posttraumatic stress symptoms
dPTCs = dysfunctional posttraumatic cognitions: PC = permanent and disturbing change, SW = fragile person in a scary world.
fPTCs = functional posttraumatic cognitions. PTE = potentially traumatic event. PTSS = posttraumatic stress symptoms.
Controlled for age, sex, trauma history, and hospitalisation. Only significant regression paths are shown. ***p<.001, **p<.01, *p<.05.

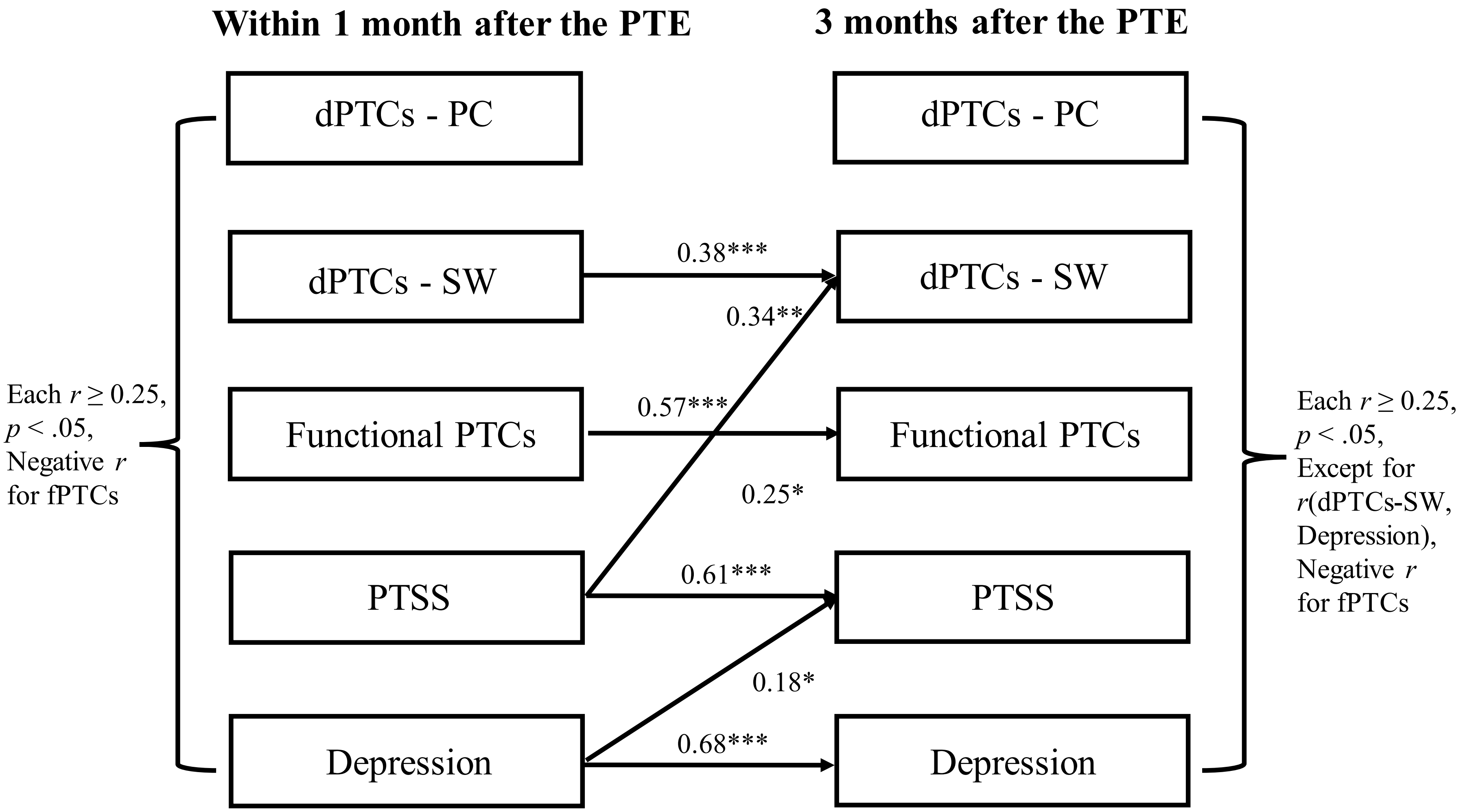

Figure S.3.4. Cross-lagged panel analysis of the longitudinal associations of posttraumatic cognitions with posttraumatic stress symptoms and depression
dPTCs = dysfunctional posttraumatic cognitions: PC = permanent and disturbing change, SW = fragile person in a scary world.
fPTCs = functional posttraumatic cognitions. PTE = potentially traumatic event. PTSS = posttraumatic stress symptoms.
Controlled for age, sex, trauma history, and hospitalisation. Only significant regression paths are shown. ***p<.001, **p<.01, *p<.05.

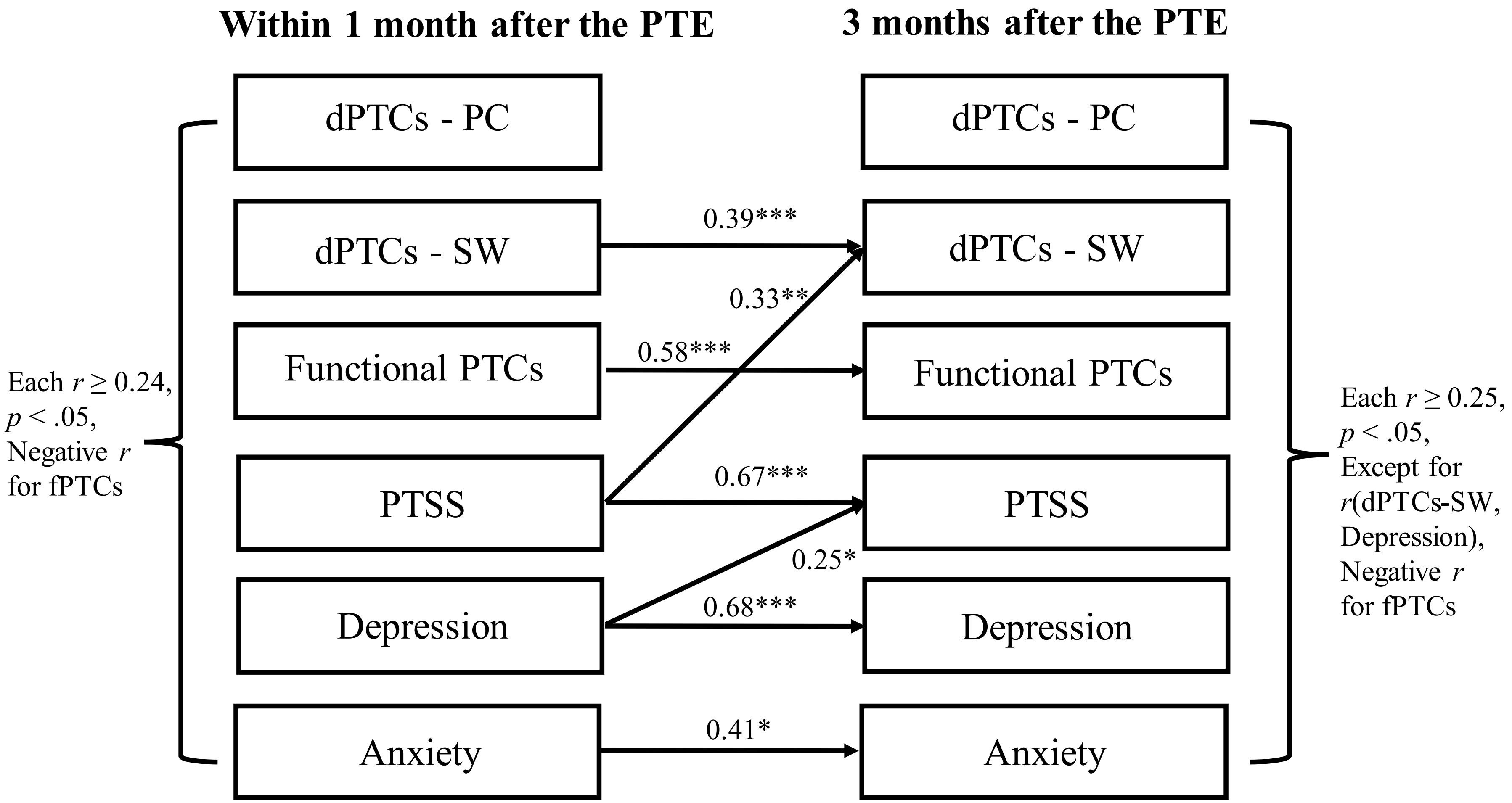

Figure S.3.5. Cross-lagged panel analysis of the longitudinal associations of posttraumatic cognitions with internalising symptoms
dPTCs = dysfunctional posttraumatic cognitions: PC = permanent and disturbing change, SW = fragile person in a scary world.
fPTCs = functional posttraumatic cognitions. PTE = potentially traumatic event. PTSS = posttraumatic stress symptoms.
Controlled for age, sex, trauma history, and hospitalisation. Only significant regression paths are shown. ***p<.001, **p<.01, *p<.05.
